# Self-Calibrated Double Luminescent Thermometers Through Upconverting Nanoparticles

**DOI:** 10.3389/fchem.2019.00267

**Published:** 2019-04-18

**Authors:** Carlos D. S. Brites, Eduardo D. Martínez, Ricardo R. Urbano, Carlos Rettori, Luís D. Carlos

**Affiliations:** ^1^Physics Department and CICECO-Aveiro Institute of Materials, University of Aveiro, Aveiro, Portugal; ^2^“Gleb Wataghin” Institute of Physics (IFGW), University of Campinas (UNICAMP), Campinas, Brazil; ^3^Center for Natural and Human Sciences, Universidade Federal do ABC, Santo André, Brazil

**Keywords:** luminescence, double thermometers, upconverting nanoparticles, primary thermometry, self-referenced thermometry, polymer nanocomposites

## Abstract

Luminescent nanothermometry uses the light emission from nanostructures for temperature measuring. Non-contact temperature readout opens new possibilities of tracking thermal flows at the sub-micrometer spatial scale, that are altering our understanding of heat-transfer phenomena occurring at living cells, micro electromagnetic machines or integrated electronic circuits, bringing also challenges of calibrating the luminescent nanoparticles for covering diverse temperature ranges. In this work, we report self-calibrated double luminescent thermometers, embedding in a poly(methyl methacrylate) film Er^3+^- and Tm^3+^-doped upconverting nanoparticles. The Er^3+^-based primary thermometer uses the ratio between the integrated intensities of the ^2^${\text{H}}_{11/2}\to^{4}$I_15/2_ and ^4^${\text{S}}_{3/2}\to^{4}$I_15/2_ transitions (that follows the Boltzmann equation) to determine the temperature. It is used to calibrate the Tm^3+^/Er^3+^ secondary thermometer, which is based on the ratio between the integrated intensities of the ^1^${\text{G}}_{4}\to^{3}$H_6_ (Tm^3+^) and the ^4^${\text{S}}_{3/2}\to^{4}$I_15/2_ (Er^3+^) transitions, displaying a maximum relative sensitivity of 2.96% K^−1^ and a minimum temperature uncertainty of 0.07 K. As the Tm^3+^/Er^3+^ ratio is calibrated trough the primary thermometer it avoids recurrent calibration procedures whenever the system operates in new experimental conditions.

## Introduction

Lanthanide-doped upconversion materials have been extensively investigated since the 1960s and displaying numerous applications due to its exceptional photophysical properties, including narrow emission lines, large anti-Stokes shift, long lifetimes, low background autofluorescence, and low toxicity (Bettinelli et al., 2015; Savchuk et al., 2018; Brites et al., 2019). The development of synthesis strategies for nanomaterials enabled a complete upconversion nanomaterials engineering, allowing the precise control of composition, morphology, size, crystalline structure, and surface chemistry (Chen et al., 2014; Wen et al., 2018).

One of the most promising applications of upconverting materials is luminescence thermometry, in which changes in photophysical properties of a material are converted into absolute temperature (Vetrone et al., 2010; Brites et al., 2012; Jaque and Vetrone, 2012). In the last decade, inorganic compounds doped with trivalent lanthanide ions (Ln^3+^) have been broadly studied as reliable ratiometric luminescent thermometers. The energy level structure of these ions allows to work in the so-called transparency biological windows (Hemmer et al., 2016) in which the tissues' absorption is minimized. The Yb^3+^/Er^3+^ couple is the most investigated upconverting dopant for a wide variety of applications, being used for bioimaging (Mader et al., 2010), photothermal therapy (Cheng et al., 2012), and for fundamental studies (Brites et al., 2016b). Albeit other approaches have been reported (Rai and Rai, 2007; Gálico et al., 2017; Brites et al., 2019), it is recognized that the wise approach for upconversion nanothermometry is based on the ratio between the integrated emission intensities of two thermally-coupled transitions. Indeed, when the emission arises from two transitions ascribed to the same emitting center with integrated intensities *I*_1_ and *I*_2_, originated in emitting levels |1> and |2> separated in energy by a Δ*E* value between 200 and 2,000 cm^−1^ (defined as thermally-coupled levels), the levels' population is governed by Boltzmann statistics and the thermometric parameter Δ is given by:

(1) $$\begin{array}{l} \Delta =\frac{I_{2}}{I_{1}}=B\exp\left(-\frac{\Delta E}{k_{B}T}\right) \end{array}$$

where *k*_B_ is the Boltzmann constant, and the pre-exponential factor *B* is dependent on the degeneracies, branching ratios, spontaneous absorption coefficients, and frequencies of the *I*_1_ and *I*_2_ transitions (Brites et al., 2019).

In terms of calibration features, the thermal probes can be sorted as secondary and primary thermometers. While for the formers a calibration procedure is mandatory, the second kind of thermometers allow the temperature determination based on an equation of state that depends only on the material's parameters (without demanding any calibration). Recently, it has been demonstrated that any upconverting thermometer based on thermally-coupled energy levels is intrinsically a primary thermometer governed by an equation of state outcoming from Equation 1 in which the pre-exponential factor *B* is rewritten in terms of a known temperature value (*T*_0_) and the corresponding ratio of intensities (Δ_0_) (Balabhadra et al., 2017):

(2) $$\begin{array}{l} \frac{1}{T}=\frac{1}{T_{0}}-\frac{k_{B}}{\Delta E}\ln\left(\frac{\Delta }{\Delta_{0}}\right) \end{array}$$

The seminal example of an upconverting luminescent primary thermometer is based on the integrated emission intensities arising from the ^2^${\text{H}}_{11/2}\to^{4}$I_15/2_ (*I*_H_) and ^4^${\text{S}}_{3/2}\to^{4}$I_15/2_ (*I*_S_) Er^3+^ transitions (Balabhadra et al., 2017). If, by one hand, the self-calibration of primary thermal probes is a great benefit (see further discussion in Brites et al., 2019), on the other hand, the typical relative thermal sensitivity *S*_r_ values of primary thermometers (Supplementary Materials) are limited to values of the order of 1.0%·K^−1^. Contrarily, secondary thermometers can render higher values (typically *S*_r_ >3.0%·K^−1^) (Marciniak et al., 2017; Brites et al., 2018, 2019). However, a new calibration is mandatory whenever secondary thermometers operate in a distinct medium and this is a serious implementation bottleneck for these devices. Thus, the wisest combination should provide the possibility of predicting the temperature through a primary thermometer displaying simultaneously a performance larger than that typical of primary thermometers.

Despite the large number of reported luminescent thermometers based on a ratio of intensities from two Ln^3+^ ions (the so-called dual-center thermometers; Brites et al., 2016a, 2019), very few are examples of double thermometers, in which the temperature is extracted from two distinct thermometric parameters. Up to now, and as far as we know, double luminescent thermometers combining two different emitting centers in the same nanostructure were reported only using Ln^3+^-doped core@shell upconverting nanoparticles (UCNPs), in all the cases through intensity ratios (Marciniak et al., 2016; Skripka et al., 2017; Martínez et al., 2019a). Marciniak et al. combined in the same UCNP one thermometer using the Er^3+^ emission in the Yb^3+^/Er^3+^ core with a second one using the Nd^3+^ downshifting emission in the Yb^3+^/Nd^3+^ shell. The nanostructure was excited at 808 nm, resonantly with the ^4^${\text{I}}_{9/2}\to^{4}$F_5/2_ Nd^3+^ transition, and a non-radiative deactivation process populated the metastable ^4^F_3/2_ state followed by sequential Nd^3+^-to-Yb^3+^ and Yb^3+^-to-Er^3+^ energy transfer processes allowing Er^3+^ upconversion emission (^4^${\text{S}}_{3/2}\to^{4}$I_15/2_ and ^2^${\text{H}}_{11/2}\to^{4}$I_15/2_) in the green spectral range (Marciniak et al., 2016). Skripka et al. further developed the same concept exciting Nd^3+^/Ho^3+^ and Nd^3+^/Er^3+^ ion pairs in UCNPs upon 800 nm. Like in the previous example, the system was excited through the Nd^3+^ ions followed by sequential Nd^3+^-to-Yb^3+^ and Yb^3+^-to-Ho^3+^ (^5^I_6_ level emitting in the 1,180–1,340 nm spectral range) or Yb^3+^-to-Er^3+^ (^4^I_11/2_ and ^4^I_13/2_ states emitting in the 1,340–1,550 nm spectral range) (Skripka et al., 2017). More recently, we described a set of electrothermal devices combining Yb^3+^/Er^3+^ and Yb^3+^/Tm^3+^-doped UCNPs of distinct sizes deposited on the top of a silver nanowires network to determine the temperature using a double thermometer combining Tm^3+^ and Er^3+^ emissions (Martínez et al., 2019a).

Here we extend the concept of this later article reporting in more detail how Yb^3+^/Er^3+^- and Yb^3+^/Tm^3+^-doped UCNPs of distinct sizes embedding in poly(methyl methacrylate) (PMMA) films can be used to fabricate self-calibrated double luminescent thermometers. Moreover, the particles' dispersion is enhanced relatively to what was published in that previous work by embedding them into polymer films (see Martínez et al., 2019b for details). This permits to study the effect on the thermometers' figures of merit of combining mixtures of UCNPs with distinct sizes (e.g., large-sized Er^3+^- and small-sized Tm^3+^-doped UCNPs and small-sized Er^3+^- and large-sized Tm^3+^-doped UCNPs).

The self-referenced nanocomposites include a luminescent primary thermal probe operating based on the ratio between the integrated intensities of the ^2^${\text{H}}_{11/2}\to^{4}$I_15/2_ and ^4^${\text{S}}_{3/2}\to^{4}$I_15/2_ Er^3+^ transitions and a secondary thermometer that uses the ratio between the integrated intensities of the ^1^${\text{G}}_{4}\to^{3}$H_6_ (Tm^3+^) and the ^4^${\text{S}}_{3/2}\to^{4}$I_15/2_ (Er^3+^) transitions. The primary thermometer is used to calibrate the secondary one (that display a higher relative thermal sensitivity and a lower temperature uncertainty), avoiding recurrent and time-consuming calibration procedures whenever the system operates in new experimental conditions. The temperature prediction in primary thermometers allows to sign changes in transitions' intensity decoupling temperature-induced changes from others resulting from distinct stimuli (*viz*. pressure, stress, etc.). Moreover, our approach of incorporating a primary self-reference thermometer is a clear step-forward toward the general implementation of luminescent thermometers as it allows the systems to be calibrated even when the conventional calibration procedure cannot be executed, as for instance, when the probes are embedded in living cells (Brites et al., 2019).

## Experimental Section

### Materials and Synthesis

The synthesis of the UCNPs and composite films was made accordingly to the procedures presented elsewhere (Martínez et al., 2018, 2019b), as detailed in Supplementary Materials. Table 1 summarizes the composition of the films.

**Table 1:** Nomenclature, nominal composition, and particle size ± std (determined using the TEM/SEM images) of the UCNPs embedded in the fabricated composite films.

**Composite film**	**Composition (nanoparticles)**	**Size (nm)**	**Representations**
C_1_	NaY_0.695_Yb_0.300_Tm_0.005_F_4_	(400 ± 15) × (120 ± 10)	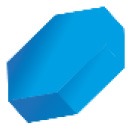
	NaGd_0.78_Yb_0.20_Er_0.02_F_4_@NaGdF_4_	11.0 ± 1.4	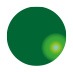
C_2_	NaGd_0.695_Yb_0.300_Tm_0.005_F_4_@NaGdF_4_	8.8 ± 0.8	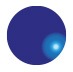
	NaY_0.78_Yb_0.20_Er_0.02_F_4_	(300 ± 8) × (160 ± 6)	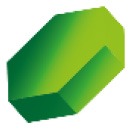

### Operating Procedure for Temperature Calibration

The composite film's temperature is controlled by a Kapton thermofoil heater (Minco) in thermal contact with the films and is determined by a thermocouple (I620–20147, VWR), also in thermal contact with the films, with an accuracy of 0.1 K. A continuous wave laser diode (980 nm, 3 × 10^4^ W·m^−2^) is used to excite the films and the upconversion emission is collected and guided to the detector (Maya 2000 Pro, Ocean Optics) through a QP450-1-XSR optical fiber (Ocean Optics). The emission spectra are subsequently post-processed using a MatLab® routine to calculate the Er^3+^ and Tm^3+^ transitions' integrated areas and the corresponding error values, as already reported (Brites et al., 2016a).

**Figure 1:**
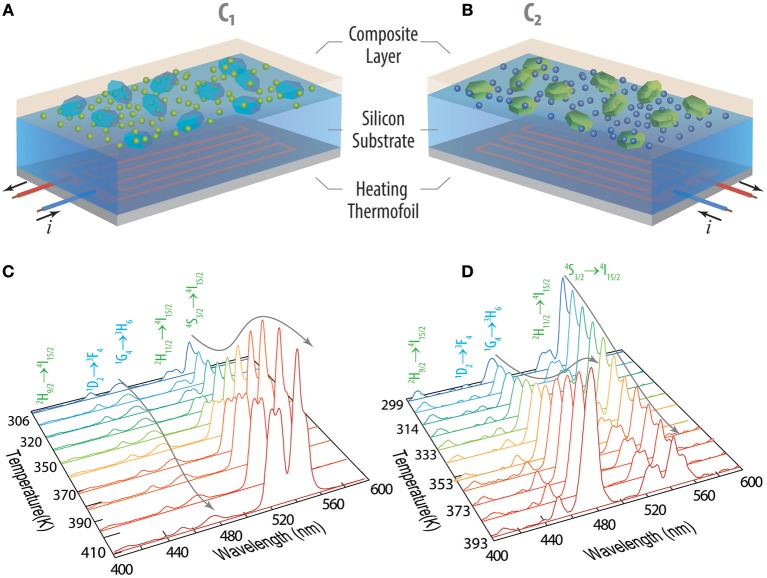
Schematic representation of the nanocomposites **(A) C**_**1**_ and **(B) C**_**2**_. The electrical current flowing in the heating thermofoil is used to control the local temperature of the UCNPs in the composite layer. The temperature dependent emission spectra upon 980 nm excitation is presented for **(C) C**_**1**_ and **(D) C**_**2**_ within the 299–410 K range. The Er^3+^ and Tm^3+^ transitions are depicted in green and blue, respectively.

The intensity-to-temperature calibration procedure is done stepping the temperature in the 299–410 K range, placing the composite films in thermal contact with the temperature controller (Figures 1A,B) during 5 min for each temperature step, and collecting the emission spectra. The measured temperature (using the thermocouple in direct contact with the sample's surface) is compared to the predicted temperature using Equation (2). In between the temperature steps, the temperature is stabilized for 15 min and, then, all the calibration procedure takes ~3 h per sample. The validity of the temperature measurements performed by the secondary thermometer self-referenced using the temperature calculated by the primary one (through Equation 2) was tested imposing a sharp temperature increase in the composite films (initially at room temperature), recording continuously the time-dependent upconversion emission spectra (during 200 s), and calculating the integrated areas of the Er^3+^ and Tm^3+^ transitions and the corresponding temperature values.

## Results and Discussion

The temperature dependent emission spectra of **C**_**1**_ and **C**_**2**_ present the characteristic narrow emission lines ascribed to the Er^3+^ and Tm^3+^ intra-4f transitions upon 980 nm excitation (power density 3 × 10^4^ W·m^−2^, Figures 1C,D). As expected, the emission intensity is thermally quenched for large-sized UCNPs and thermally enhanced for small-sized UCNPs (Martínez et al., 2019a). For the large-sized nanoparticles, the increase of temperature induces a systematic decrease of the emission intensity. All transitions suffer thermal quenching upon temperature increase, although in distinct extents. It is well-known that the thermal quenching in micro-sized particles and bulk upconverting materials has been frequently attributed to multi-phonon non-radiative relaxation mechanisms, resulting in higher decay probabilities, and, thus, the observed trends are expected (Shen et al., 2010; Yu et al., 2016). On the contrary, intensity enhancement (or reverse quenching) with the increasing temperature observed for small-sized UCNPs is in agreement with the findings firstly reported by Jiang's group (Li et al., 2014, 2015; Shao et al., 2015, 2017), and more recently by Zhou et al. (2018). The later work explained the observed thermal enhancement of the intensity for small-sized UCNPs by heat-favorable phonons existing at the surface of UCNPs that compensate the thermal quenching, favoring the energy transfer from sensitizers to activators to pump-up the intermediate excited-state upconversion process. The authors argued that the oxygen moiety chelating the Yb^3+^ ions is the key underpinning this enhancement. However, a definitive physical mechanism that fully explains this surface phonon-assisted energy transfer mechanism remains inconclusive ((Liang and Liu, 2018);Martínez et al., 2019a).

**Figure 2:**
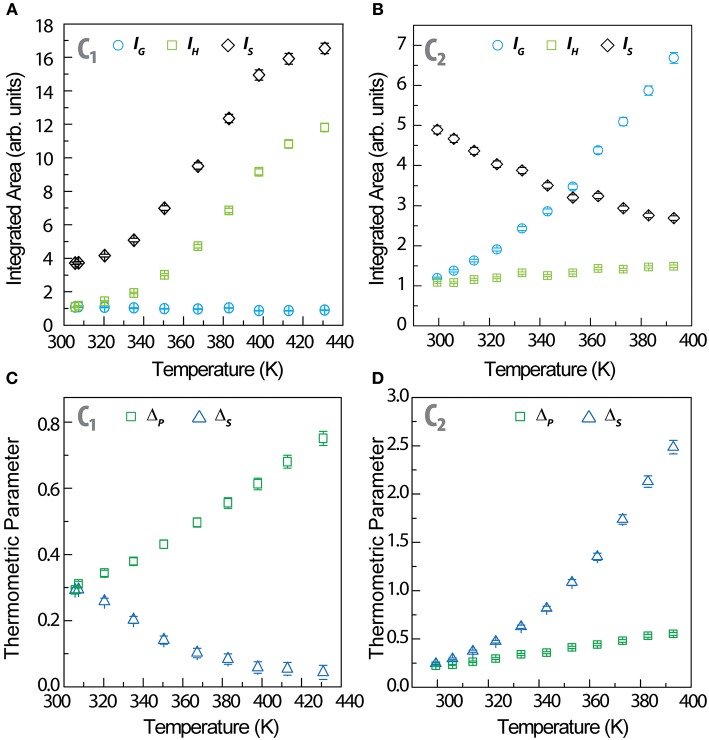
Temperature dependence of the integrated areas of *I*_H_, *I*_S_ (Er^3+^) and *I*_G_ (Tm^3+^) for **(A) C**_**1**_ and **(B) C**_**2**_. The corresponding Δ_P_ and Δ_S_ thermometric parameters are presented in **(C)** and **(D)**, respectively.

Analyzing the temperature dependence of the integrated intensities of the ^2^${\text{H}}_{11/2}\to^{4}$I_15/2_ (*I*_H_), ^4^${\text{S}}_{3/2}\to^{4}$I_15/2_ (*I*_S_), and ^1^${\text{G}}_{4}\to^{3}$H_6_ (*I*_G_) transitions upon increasing the temperature, we observe an increase of *I*_S_ and *I*_H_ and a marginal decrease of *I*_G_ (Figure 2A) for **C**_**1**_, whereas the opposite occurs for **C**_**2**_: *I*_S_ decreases, *I*_H_ is roughly constant and *I*_G_ increases (Figure 2B). The thermometric parameter of the Er^3+^-based primary thermometer is defined as Δ_*P*_ = *I*_H_/*I*_S_, whereas that of the Er^3+^/Tm^3+^-based secondary thermometer is defined as Δ_*S*_ = *I*_G_/*I*_S_. Observing the temperature dependence of the intensity ratios from the primary and secondary thermometers, we notice a clear steep increase in the Δ_*S*_ ratio compared to the Δ_*P*_ one (Figures 2C,D). This indicates an improved in the *S*_r_ values for the dual-center secondary thermometer, in line to what is observed before, as detailed next.

**Figure 3:**
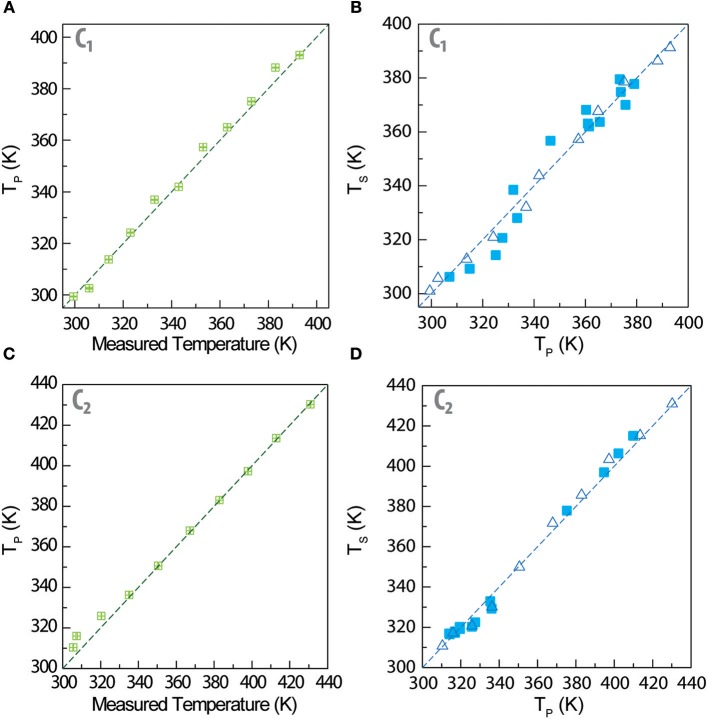
Temperature dependence of the relative thermal sensitivity of primary and secondary thermometers for **(A) C**_**1**_ and **(B) C**_**2**_. **(C,D)** Temperature dependence of the corresponding temperature uncertainties, respectively.

The maximum relative thermal sensitivity of the primary thermometers in **C**_**1**_ and **C**_**2**_ (1.31 and 1.19%·K^−1^, respectively) (Figures 3A,B) and the minimum temperature uncertainty (0.15 and 0.18 K, respectively, all at 300 K) (Figures 3C,D) are comparable to the values reported for Er^3+^- based thermometers (Brites et al., 2019). Moreover, the relative thermal sensitivity of the primary thermometers (Figures 3A,B) is independent on nanoparticle's dimensions, in line with our previous observations (Balabhadra et al., 2017; Brites et al., 2019). The relative thermal sensitivity of the secondary thermometers presents the typical functional forms observed for dual-center luminescent thermometers (Figures 3A,B) (Brites et al., 2016a) with maximum values of 2.96%·K^−1^ (**C**_1_ at 300 K) and 2.28%·K^−1^ (**C**_**2**_ at 350 K), corresponding to minimum temperature uncertainties of 0.07 K at 300 K (**C**_**1**_) and 0.09 K at 350 K (**C**_**2**_). There is an improvement in the relative thermal sensitivity values by a factor of up to 2.3 (in **C**_**1**_), relatively to the same parameters calculated for the primary thermometer and the most advantageous combination of UCNPs is the mixture of large-sized Tm^3+^ -doped and small-sized Er^3+^ -doped UCNPs (Figure 3).

Comparing the performance of **C**_**1**_ with those of other double luminescent thermometers in the literature, we conclude that the sensitivity values are the largest reported so far. Whereas, the system reported by Marciniak et al. presents a maximum relative sensitivity of 2.1%·K^−1^ at 370 K (Marciniak et al., 2016) that reported by Skripka et al. don't present values higher than 1.1%·K^−1^ (Skripka et al., 2017), meaning that the composite films reported here presents an increase of 40% with respect to these results. Moreover, in comparison with the system developed by us using the same design principle (Martínez et al., 2019a), the nanocomposites studied here present comparable sensitivity values for the primary thermometer and about half of the sensitivity value for the secondary one. We ascribe these differences in performance to the encapsulation of the UCNPs in the PMMA that resulted in a smoother temperature enhancement of the Tm^3+^ emission of small-sized nanoparticles, in comparison with that observed previously for similar nanoparticles deposited directly on silver nanowires and exposed to the air (Martínez et al., 2019a). This is a remarkable relative thermal sensitivity tuning-up that we will exploit in a future work.

As the nanocomposite films combining the Er^3+^ and Tm^3+^ UCNPs permit to define one primary (Δ_P_) and another secondary thermometric parameter (Δ_S_), in what follows we show how that the first parameter (that follows Equation 2, section Er^3+^-based primary thermometers) can be used to calibrate the secondary thermometer (section Self-referenced Er^3+^/Tm^3+^ secondary thermometers).

### Er^3+^-Based Primary Thermometers

Despite the opposite behavior of the temperature dependence of the integrated areas of the Er^3+^ transitions for small (<10 nm) and large-sized (>100 nm) UCNPs, the ratio Δ_*P*_ = *I*_H_/*I*_S_ always grows with the increasing temperature, irrespectively of the nanoparticle size and morphology (nanospheres or nanocrystals). To predict the temperature through Equation 2, the Δ*E* and Δ_0_ values must be calculated for each UCNP by independent measurements (and not as fitted parameters of Equation 1). There are distinct strategies reported in the literature for extracting these parameters (Brites et al., 2019). The energy gap Δ*E* is evaluated deconvoluting the emission spectra at room temperature by a set of Gaussian peaks and evaluating the position of the ^2^${\text{H}}_{11/2}\to^{4}$I_15/2_ and ^4^${\text{S}}_{3/2}\to^{4}$I_15/2_ transitions' barycenter (Supplementary Figure 1), whereas Δ_0_ is the thermometric parameter corresponding to the temperature *T*_0_. It can be evaluated measuring the excitation power dependence of the thermometric parameter (Debasu et al., 2013), or just assuming the initial value of Δ_*P*_ at *T*_0_ (room-temperature), when there is no excitation laser-induced heating (low excitation power density values 3 × 10^4^ W·m^−2^). Notice the dissimilar *T*_0_ values are consequence of distinct operating ambient conditions during the spectra acquisition. The Δ*E* and Δ_0_ parameters for large- and small-sized Er^3+^-doped UCNPs are presented in Table 2. The calculated Δ*E* values are similar for both composite films within the corresponding experimental errors and are in good agreement with those reported in the literature (Carnall et al., 1977).

**Table 2:** Δ*E* (cm^−1^), Δ_0_ and *T*_0_ (K) values of the primary thermometer in C_1_ and C_2_.

**Composite film**	**ΔE**	**Δ_0_**	***T*_**0**_**
C_1_	780 ± 15	0.222 ± 0.006	299.4 ± 0.1
C_2_	749 ± 15	0.293 ± 0.008	305.6 ± 0.1

**Figure 4:**
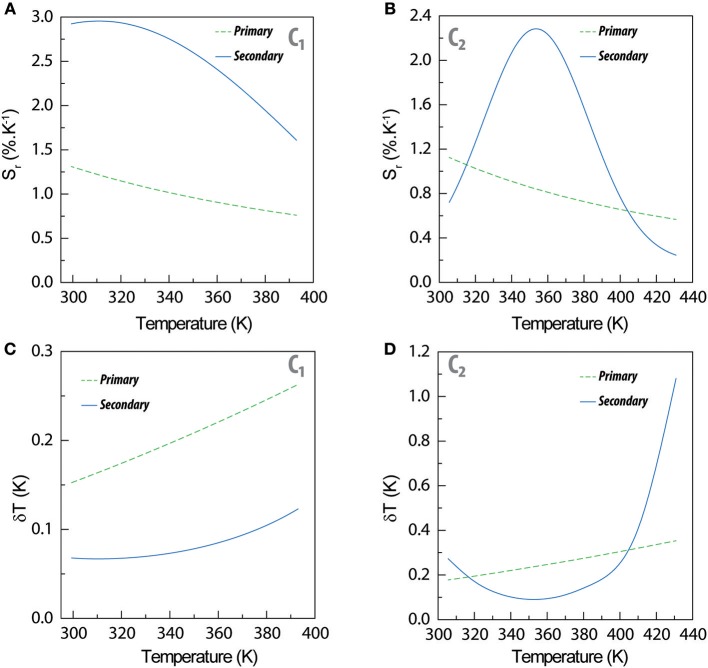
Temperature measured by a thermocouple in contact with the composite films (*x*) vs. the temperature calculated using Equation 2 (*T*_p_, *y*) for **(A) C**_**1**_ and **(B) C**_**2**_. Calculated temperature *T*_p_ (*x*) vs. calculated temperature *T*_s_ (*y*) using the calibration curve of the Er^3+^/Tm^3+^ secondary thermometer (*T*_s_, y) for **(B) C**_**1**_ and **(D) C**_**2**_. In (**B**,**D)**, the open and solid symbols correspond to the calibration points and validity checks, respectively, as detailed in Experimental section. The straight lines correspond to *y* = *x* in all the plots.

In Figures 4A,C we compare the temperature calculated through Equation (2) (*T*_p_) with that measured by a thermocouple in direct contact with the sample's surface. The remarkable agreement observed in both nanocomposites demonstrates that the primary thermometric parameter Δ_P_ permits to determine the temperature of the films using Equation (2), in excellent agreement with the values that are measured by a control temperature probe in contact with the nanocomposite's surface. These results validate the use of the ratio of intensities of the ^2^${\text{H}}_{11/2}\to^{4}$I_15/2_ and ^4^${\text{S}}_{3/2}\to^{4}$I_15/2_ Er^3+^ transitions for primary thermometry, as it has been systematically observed since the initial purpose of some of us (Balabhadra et al., 2017; Brites et al., 2019). Moreover, the relative thermal sensitivity of the primary thermometers in **C**_**1**_ and in **C**_**2**_ are only determined by the Δ*E* values (Equation 1 and Supplementary Equation 1) listed in Table 2, and that depend on the size and on the composition of the Er^3+^-doped UCNPs. The maximum *S*_r_ values are comparable to those reported for other luminescent thermometers based on Er^3+^-doped UCNPs (Brites et al., 2019).

### Self-Referenced Er^3+^/Tm^3+^ Secondary Thermometers

The secondary luminescent thermometer based on the Δ_S_ parameter determines the temperature (denoted by *T*_S_) using the phenomenological calibration curve described by Supplementary Equation 3 and presented in Supplementary Figure 2. In Figures 3B,D that temperature *T*_s_ is compared with that extracted from Equation (2) using the Δ_P_ values (*T*_P_). It is remarkable that we calculate similar temperature values (*T*_P_ and *T*_S_, based on the two thermometric parameters Δ_P_ and Δ_S_), that agree very well with the values measured by the thermocouple in contact with the nanocomposite's surface. The temperature predicted through Equation 2 (primary thermometer) is used to calibrate the secondary thermometer (presenting a higher relative thermal sensitivity) enabling its self-referencing and not requiring the presence of an external thermocouple to set the calibration temperature during a large time period (3 h, as mentioned above).

To exemplify that the nanocomposite's temperature control can waive the record of an intensity-to-temperature calibration curve, we design a validity check experiment that consists in record the time-evolution of the emission spectra during 200 s, calculating the corresponding Δ_*P*_ and Δ_*S*_ values (the temporal evolution of the emission spectra is presented in Supplementary Figure 2). For each emission spectrum, we convert Δ_P_ into temperature using Equation (2) (Figures 3B,D) and Δ_S_ into temperature using Supplementary Equation 3. The results are presented as solid symbols in Figures 3B,D. We observe an excellent agreement between the conventional calibration and the validity check points, meaning that both calibration procedures are similar within the experimental error. Moreover, the temperature values calculated from both intensity ratios are statistically similar to those measured with the conventional calibration procedure, with an incredible gain in terms of time efficiency, because the heating ramp recording is more than 50 times faster than the conventional temperature stepping procedure.

The step forward presented in this work relatively to what was reported previously by us (Martínez et al., 2019a) is the comparison of the thermometric performances of the secondary thermometers formed by mixtures of large-sized Tm^3+^- and small-sized Er^3+^-doped UCNPs (**C**_**1**_) with small-sized Tm^3+^- and large-sized Er^3+^-doped UCNPs (**C**_**2**_). As expected, the functional form of *S*_r_ is the same for both composites, and for UCNPs deposited directly over a Ag-nanowires network (Martínez et al., 2019a), because it results from the phenomenological function used for fitting Δ_S_, Supplementary Equation 3 which is the same in all the three examples. Moreover, in the temperature range studied the S_r_ values of **C**_**1**_ and **C**_**2**_ varies, respectively, between 1.65 and 2.93%·K^−1^ and 0.72 and 2.28%·K^−1^ (Figures 4A,B). These values are consistent with the changes on the integrated areas presented in Figures 2A,B showing that the performance of the secondary thermometer is essentially determined by the temperature enhancement observed in the integrated areas of the transitions of the small UCNPs. Furthermore, the combination of small Tm^3+^-doped and large Er^3+^-doped UCNPs resulted in a narrower *S*_r_ peak for **C**_**2**_ (in comparison with **C**_**1**_), that in that is comparable with that reported by us previously for UCNPs deposited directly over a Ag-nanowires network (Martínez et al., 2019a). The incorporation of the UCNPs into the PMMA film resulted in a decrease of the maximum *S*_r_ value and in the temperature at which it occurs (Supplementary Figure 4), in comparison with our previous work (Martínez et al., 2019a). This is consequence of the thermal dependence of the integrated area of the ^1^${\text{G}}_{4}\to^{3}$H_6_ transition, that growths about seven times for **C**_**2**_ whereas in our previous work it increases about 18 times in a comparable temperature range (Martínez et al., 2019a). Thus, we conclude that the transitions originated in the small-sized Tm^3+^ particles are determining the performance of these devices (Figure 4). The observed changes in the integrated areas of small -sized UCNPs resulting from their embedding into PMMA are still not entirely understood, needing further experimental evidences (specially in what concerns the incorporation of UCNPs in other hosts). Work is in progress along this research line.

## Conclusions

In this work, we combined a primary thermometer and a secondary thermometer rendering to self-referenced double thermometric systems in the same composite film with relative thermal sensitivity comparable with the largest ones reported yet for secondary thermometers. To illustrate this concept, Ln^3+^-doped NaYF_4_ and NaGdF_4_ nanoparticles (Ln = Yb, Er, Tm) of distinct sizes were embedded in two PMMA films. The nanocomposites' inner temperature reference is the primary thermometer based on the ^2^${\text{H}}_{11/2}\to^{4}$I_15/2_ (*I*_H_) and ^4^${\text{S}}_{3/2}\to^{4}$I_15/2_ (*I*_S_) Er^3+^ transitions, univocally assigning each emission spectrum to the corresponding temperature using Boltzmann equation. We attest that the system is a primary thermometer comparing the predicted and measured temperature values and observing an excellent agreement between both. The secondary thermometer is based on the temperature dependence of a ratio of intensities involving one emission intensity that is thermally quenched (large-sized UCNPs) and another one that is thermally enhanced (small-sized UCNPs). The maximum relative thermal sensitivity of this thermometer is 2.96%·K^−1^ and the minimum temperature uncertainty is 0.07 K (both at 300 K), among the highest performance values reported so far for luminescent dual thermometers. Moreover, the maximum *S*_r_ value corresponds to a 2.3-fold improvement, with respect to the Er^3+^-based primary thermometer.

This highly sensitive thermometer can be calibrated using a conventional temperature-stepping procedure, taking a total of 3 h, or using the primary thermometer to calibrate it. We validate the resulting calibration curves recording the emission spectra in a heating ramp and observing a good agreement between the temperature values calculated from the primary and the secondary thermometers independently. Although in our previous work (Martínez et al., 2019a) we adopted this faster method to calibrate the secondary thermometer, here we demonstrate that conventional and fast calibration procedures are equivalent and, thus, the external temperature control is not mandatory to calibrate the self-referenced system taking only 200 s, that constitutes a procedure more than 50 times faster than the conventional calibration.

Finally, we stress that the procedure described here of incorporating an inner self-referenced temperature probe (Er^3+^ doped UCNPs) is general and can be applied for any system that require thermal calibration. Such dual systems present the critical advantage of the secondary thermometer being more sensitive than the primary. Since the secondary thermometer can be calibrated “*in situ*,” this avoids conventional methods of calibration, and opens the way for applications in biological media, particularly at the cell level. This strategy will certainly pave the road for the future routinely use of self-calibrated dual luminescent thermometers based on UCNPs, allowing to avoid long calibration procedures that require sophisticated temperature controllers, without sacrificing the temperature readout error.

## Data Availability

The raw data supporting the conclusions of this manuscript will be made available by the authors, without undue reservation, to any qualified researcher.

## Author Contributions

CB, LC, and EM conceived the project. EM synthesized the particles and performed all measurements. CB, EM, and LC discussed the project, analyzed the data, performed the calculations, and prepared all figures. RU and CR contributed with the experimental set-up and discussion of the project. The manuscript was written with contributions from all authors.

### Conflict of Interest Statement

The authors declare that the research was conducted in the absence of any commercial or financial relationships that could be construed as a potential conflict of interest.
