# Supplementary material for: Self-Calibrated Double Luminescent Thermometers Through Upconverting Nanoparticles
Source: Front Chem. 2019 Apr 18;7:267. doi: 10.3389/fchem.2019.00267 (PMC6482206; doi:10.3389/fchem.2019.00267)
Supplement: Supplementary file 1 [file Data_Sheet_1.pdf]

Supplementary Materials

Self-calibrated dual luminescent thermometers through upconverting nanoparticles

Carlos D. S. Brites[1*], Eduardo D. Martínez[2],
Ricardo R. Urbano[2], Carlos Rettori[2,3], Luís D. Carlos[1]

[1] Physics Department and CICECO-Aveiro Institute of Materials, University of Aveiro, 3810-193 Aveiro, Portugal.

[2] "Gleb Wataghin" Institute of Physics (IFGW), University of Campinas (UNICAMP), 13083-859, Campinas, SP, Brazil.

[3] CCNH, Universidade Federal do ABC (UFABC),09210-580, Santo André, SP, Brazil.

*** Correspondence:**
Carlos D.S. Brites.
carlos.brites@ua.pt

1 Materials and Synthesis 1
1.1 Materials 2
1.2 Synthesis of UCNPs 2
1.3 UCNPs-polymer nanocomposites 3
2 Quantifying the performance of a thermometer 3
3 Determining the energy separation between the $^4S_{3/2}$ and $^2H_{11/2}$ levels 4
4 Secondary thermometers calibration 5
Supplementary References 7

1 Materials and Synthesis

1.1 Materials

Sodium trifluoroacetate 98% (CAS 2923-18-4), yttrium(III) trifluoroacetate >99.99% (CAS 304851-95-4), erbium(III) oxide (CAS 12061-16-4), ytterbium(III) oxide (CAS 1314-37-0), thulium(III) oxide (CAS 12036-44-1), trifluoroacetic acid 99% (CAS 76-05-1), , oleic acid (OA, technical grade, 90%) and 1-octadecene (ODE, technical grade, 90%) were purchased from Sigma-Aldrich Co. Gadolinium(III) acetate hydrate 99.9% (CAS 100587-93-7), Cerium(III) chloride heptahydrate 99% (CAS 18618-55-8) and Poly(methyl methacrylate) (PMMA) Mw 400-550 kg·mol^{-1} (CAS 9011-14-7) were purchased from Alfa Aesar. All commercial reagents were used without further purification. Rare-earth acetates and trifluoroacetates not listed above were prepared in our laboratory using the corresponding rare-earth oxides (X_2O_3, X = Yb, Tm, Er). In a 500 mL round bottom flask containing 30–50 mL of a 50% (v/v) aqueous solution of either acetic acid or trifluoroacetic acid, 1–2 g of each oxide was added. The mixture was refluxed for 1-2 hours until the complete dissolution of the oxides resulted in a clear solution. The solution was then transferred to a Pyrex open vessel and maintained at 60 °C to evaporate the acid. The dried precipitates of acetate/trifluoroacetate salts were extracted.

1.2 Synthesis of UCNPs

Large (300 nm) pure hexagonal phase $NaYF_4$ nanoparticles co-doped with ytterbium (20%) and erbium (2%) were synthesized by the well-established thermal decomposition method of fluoroacetates, adapting protocols described by Ye and co-workers (Ye et al., 2010). In a three neck 200 mL round bottom flask containing 15 mL of ODE and 15 mL of OA, a total of 6.2 mmol of $NaCOOCF_3$, 2.6 mmol of $Yb(COOCF_3)_3$ and 0.68 mmol of $Er(COOCF_3)_3$ were added. The flask was sealed and heated under vacuum at 125 °C in order to dissolve the precursors and degas the solution. After 1 hour, a condenser was mounted, and the temperature was rapidly increased (10-20 °C·min^{-1}) up to 330 °C under an argon flux. After 30–35 minutes from the beginning of the heating stage, the reaction flask was retired from the mantle and 15 mL of ODE were added to quench the reaction. Extraction of the UCNPs was performed by adding an ethanol:hexane 4:1 v/v mixture and centrifugation for 5 minutes at 2400 rpm, corresponding to a relative centrifugal force (RCF) of 1004 RCF. The washing procedure was repeated 4 times and the UCNPs were finally dispersed in hexane with a final concentration of about 2 g·cm^{-3}.

Small size hexagonal core-UCNPs (<12 nm) were synthesized by the co-precipitation route adapting the protocols reported by Wang and co-workers (Wang et al., 2014). using rare-earth acetates as the main precursors. A total of 1 mmol of $X(COOCH_3)_3$ (X = Gd, Yb, Tm, Er) was added in a 200

mL round bottom flask containing 15 mL of ODE and 9 mL of OA. The nominal molar ratio of each system was 1:0.78:0.2:0.02 Na:Gd:Yb:Er and 1:0.695:0.3:0.005 Na:Gd:Yb:Tm. The mixture was heated up to 160 °C for 1 hour to dissolve the precursors and then cooled down to room temperature. At this point, a freshly prepared mixture containing 2.5 mL of a 1 M NaOH methanol solution and 10.1 mL of 0.4 M NH_4F solution in methanol was rapidly injected. The flask was heated to 50 °C for 30 minutes and then sealed. The temperature was raised to 100 °C and the vacuum pump was connected. After 15 minutes, the vacuum pump was turned off, a condenser was mounted, and the temperature was increased to 280 °C under an argon flux. The flask was retired from the mantle after 1 hour and allowed to cool down to room temperature. Extraction of the UCNPs was performed by adding excess anhydrous ethanol and centrifugation using 15 mL Falcon tubes. Centrifugation was performed at 1004 RCF (2400 rpm) for 7 minutes. The precipitated UCNPs were redispersed in 4 mL of cyclohexane and ethanol was added to complete the volume. The centrifugation and washing procedure were repeated twice. Finally, the UCNPs were dispersed in 8 mL of cyclohexane. As the quantum yield of the UC process is lower for smaller particles, a core–shell structure was formed by growing a thin layer of undoped $NaGdF_4$ on the surface of the pre-synthesized core UCNPs. For this, 1 mmol of $Gd(COOCH_3)_3$ was added to 8 mL of OA and 12 mL of ODE in a 200 ml three-neck round bottom flask. The mixture was heated up to 160 °C for 1 hour and then cooled down to room temperature. Then, 6 mL of the core UCNPs colloid in cyclohexane were added. The subsequent addition of a methanol solution of NaOH and NH_4F and the following procedures were identical to those previously described for the synthesis of core nanoparticles.

1.3 UCNPs-polymer nanocomposites

For preparation of the nanocomposites, a certain amount of the colloids formed by UCNPs in cyclohexane was placed in a glass vial under a gentle flux of N_2 until complete evaporation of the solvent. The dried UCNPs were dispersed in a 2 %wt. PMMA solution in chloroform by means of vortex agitation and ultrasonic bath. The prepared nanocomposite solutions were spin-coated at 3000 rpm on pieces of silicon wafers, forming a thin uniform layer. The films were subjected to thermal pretreatment at 413 K for 30 min prior to any measurement.

2 Quantifying the performance of a thermometer

It is well established that the performance of a thermometer can be quantitatively expressed in terms of its relative thermal sensitivity (S_r), defined as (Brites et al., 2018b):

$$S_r = \frac{1}{\Delta}\left|\frac{\partial \Delta}{\partial \mathrm{T}}\right| \quad \text{(S 1)}$$

and in terms of its temperature uncertainty (δT) on particular experimental conditions (Brites et al., 2016;Brites et al., 2018a;Brites et al., 2018b):

$$\delta T = \frac{1}{S_r}\frac{\delta\Delta}{\Delta}, \quad \text{(S 2)}$$

where $\delta\Delta/\Delta$ is the relative uncertainty on the intensity ratio, estimated as 0.20 %. This is the minimum relative uncertainty in Δ achieved experimentally despite $\mathbf{C_2}$ produces lower signal to noise ratio for the same experimental conditions, since it presents the small-sized Tm^{3+}-doped UCNPs.

3 Determining the energy separation between the $^4S_{3/2}$ and $^2H_{11/2}$ levels

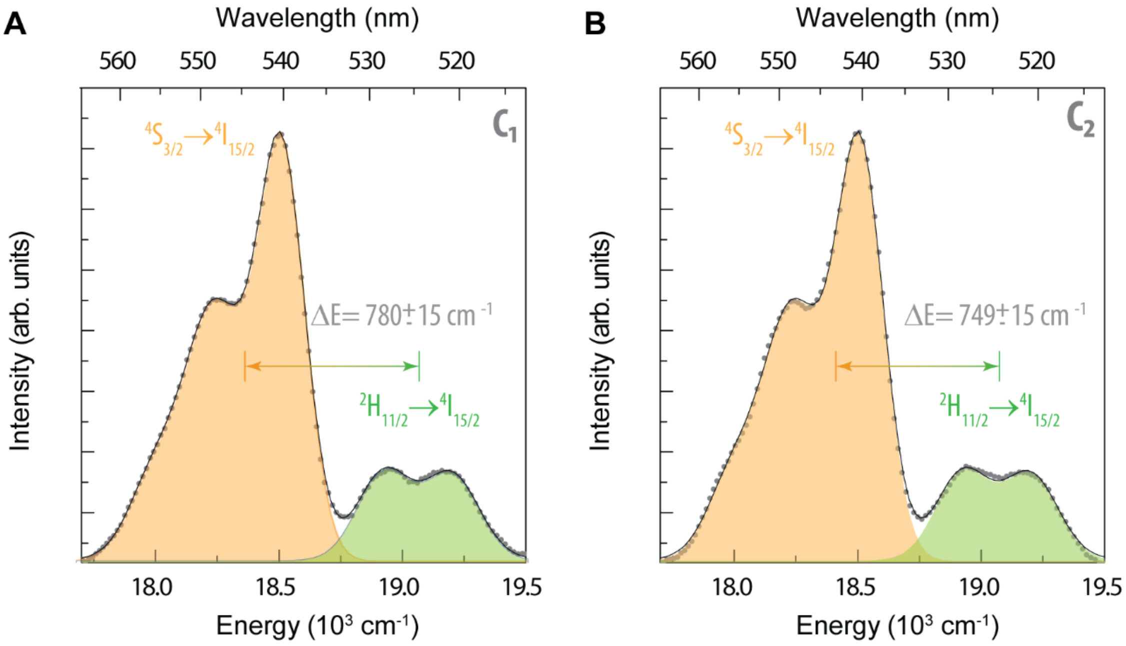

Supplementary Figure 1. Energetic separation between the emitting levels of Er^{3+} in the samples **(A)** C_1 and **(B)** C_2.

4 Secondary thermometers calibration

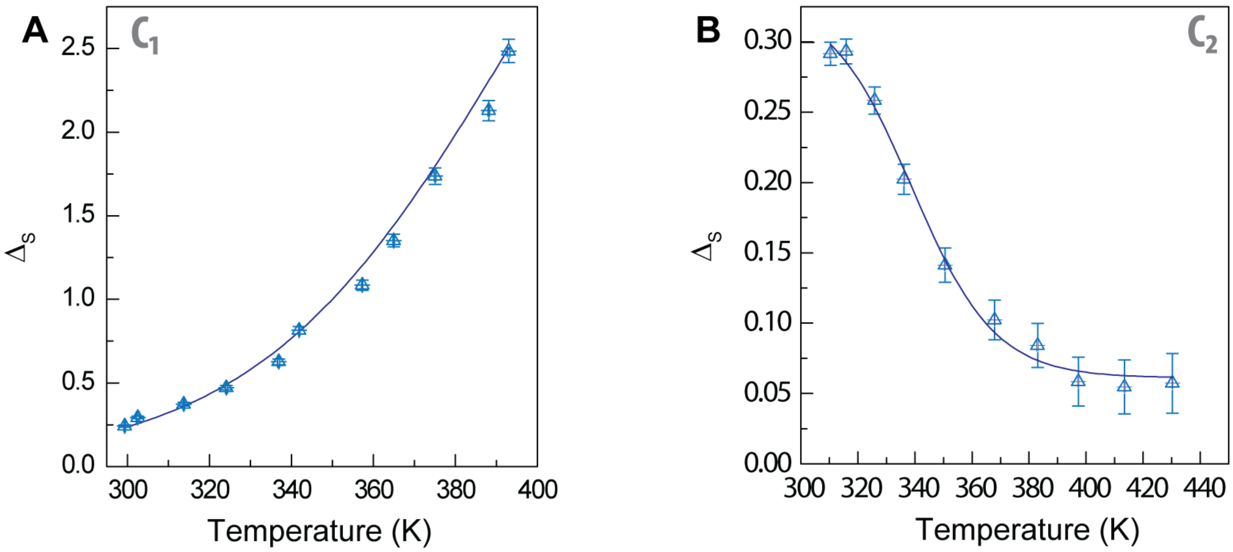

Supplementary Figure 2. Calibration curves of the secondary thermometers of **(A) C_1** and **(B) C_2**. The solid lines are the best fit of the datapoints to Eq S3 with the parameters presented in Supplementary Table 1.

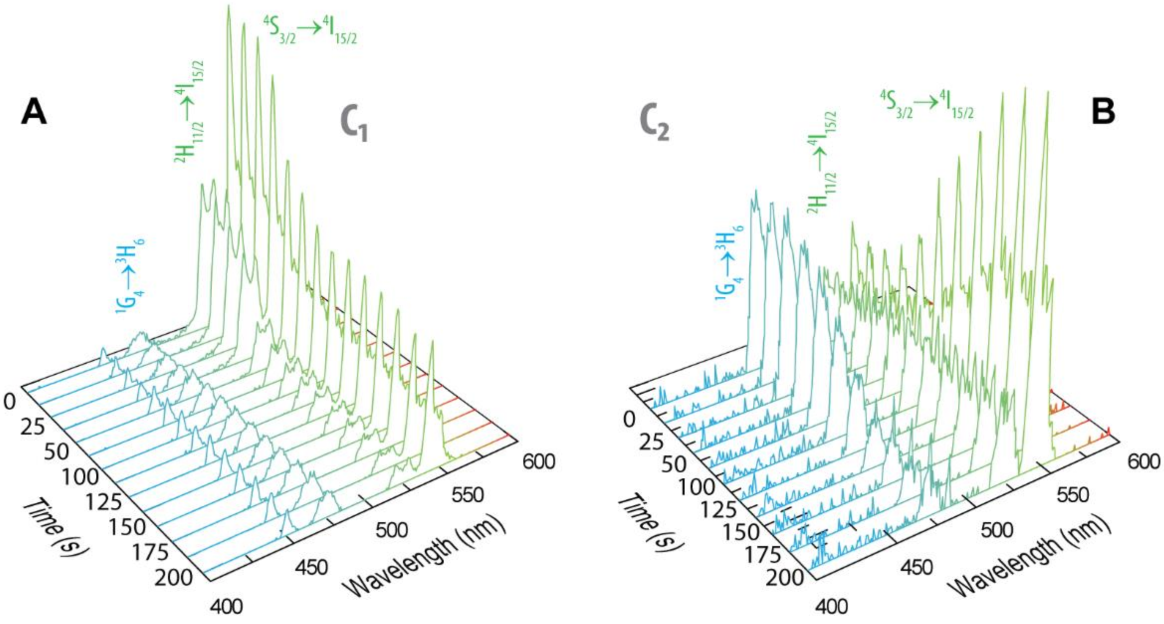

Supplementary Figure 3. Emission spectra recorded was a function of elapsed time for contact with a hot plate for **(A) C_1** and **(B) C_2**.

The phenomenological calibration curves of the secondary thermometers and are obtained by fitting the temperature dependences of Δ_S for both composite films using the OriginLab® program and a Boltzmann fitting curve (Supplementary Eq. S3). The resulting fitting parameters are listed in Supplementary Table 1.

In Eq. S3, the fitting parameters Δ_1 and Δ_2 are the limits of Δ_S for T_S<<T_m and T_S>>T_m, respectively, and T_m and ΔT are characteristic temperature values controlling the temperature dependence of Δ_S.

$$\Delta_S(\mathrm{T}) = \frac{\Delta_1 - \Delta_2}{1 + \exp\left(\frac{T_S - T_m}{\Delta T}\right)} + \Delta_2 \qquad \text{(S 3)}$$

Supplementary Table 1. Parameters of the fitting to the experimental calibration points presented in Supplementary Figure 2.

Parameter	***Composite Film***	
	**C1**	**C2**
Δ_1	*0.042±0.009*	*0.33±0.02*
Δ_2	*6.5±0.5*	*0.061±0.009*
T_m	*410±14*	*339±3*
ΔT	*32±4*	*15±3*
r^2	*0.993*	*0.991*

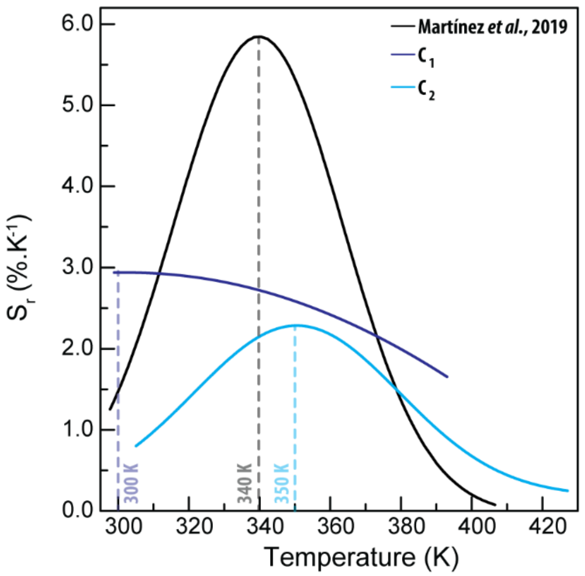

Supplementary Figure 4. Relative thermal sensitivity of **C_1** and **C_2** secondary thermometers compared with that previously reported for comparable systems (Martínez et al., 2019). The interrupted lines mark the temperature of maximum relative thermal sensitivity.

Supplementary References

Brites, C.D., Balabhadra, S., and Carlos, L.D. (2018a). Lanthanide-based thermometers: At the cutting-edge of luminescence thermometry. *Advanced Optical Materials***,** in press.

Brites, C.D., Balabhadra, S., and Carlos, L.D. (2018b). Lanthanide-Based Thermometers: At the Cutting-Edge of Luminescence Thermometry. *Advanced Optical Materials* 10.1002/adom.201801239**,** 10.1002/adom.201801239.

Brites, C.D.S., Millán, A., and Carlos, L.D. (2016). "Lanthanides in Luminescent Thermometry," in *Handbook on the Physics and Chemistry of Rare Earths*, eds. J.-C.G. Bünzli & V.K. Pecharsky. (Amsterdam: Elsevier Science, B. V.), 339-427.

Martínez, E.D., Brites, C.D.S., Carlos, L.D., García-Flores, A.F., Urbano, R.R., and Rettori, C. (2019). Electrochromic Switch Devices Mixing Small- and Large-Sized Upconverting Nanocrystals. *Advanced Functional Materials* 10.1002/adfm.201807758**,** 10.1002/adfm.201807758.
